# Cream Formulation Impact on Topical Administration of Engineered Colloidal Nanoparticles

**DOI:** 10.1371/journal.pone.0126366

**Published:** 2015-05-11

**Authors:** Benedetta Santini, Ivan Zanoni, Roberta Marzi, Clara Cigni, Marzia Bedoni, Furio Gramatica, Luca Palugan, Fabio Corsi, Francesca Granucci, Miriam Colombo

**Affiliations:** 1 NanoBioLab, Dipartimento di Biotecnologie e Bioscienze, Università degli Studi di Milano-Bicocca, Milano, Italy; 2 Division of Gastroenterology, Boston Children's Hospital, Harvard Medical School, Boston, Massachusetts, United States of America; 3 Laboratorio di Nanomedicina e Biofotonica Clinica, Fondazione Don Carlo Gnocchi ONLUS, Milano, Italy; 4 Dipartimento di Scienze Farmaceutiche, Università degli Studi di Milano, Milano, Italy; 5 Dipartimento di Scienze Biomediche e Cliniche “Luigi Sacco”, Università degli Studi di Milano, Milano, Italy; 6 Unit of Cell Signalling and Innate Immunity, Humanitas Clinical and Research Center, Rozzano, Milan, Italy; Brandeis University, UNITED STATES

## Abstract

In order to minimize the impact of systemic toxicity of drugs in the treatment of local acute and chronic inflammatory reactions, the achievement of reliable and efficient delivery of therapeutics in/through the skin is highly recommended. While the use of nanoparticles is now an established practice for drug intravenous targeted delivery, their transdermal penetration is still poorly understood and this important administration route remains almost unexplored. In the present study, we have synthesized magnetic (iron oxide) nanoparticles (MNP) coated with an amphiphilic polymer, developed a water-in-oil emulsion formulation for their topical administration and compared the skin penetration routes with the same nanoparticles deposited as a colloidal suspension. Transmission and scanning electron microscopies provided ultrastructural evidence that the amphiphilic nanoparticles (PMNP) cream formulation allowed the efficient penetration through all the skin layers with a controllable kinetics compared to suspension formulation. In addition to the preferential follicular pathway, also the intracellular and intercellular routes were involved. PMNP that crossed all skin layers were quantified by inductively coupled plasma mass spectrometry. The obtained data suggests that combining PMNP amphiphilic character with cream formulation improves the intradermal penetration of nanoparticles. While PMNP administration in living mice via aqueous suspension resulted in preferential nanoparticle capture by phagocytes and migration to draining lymph nodes, cream formulation favored uptake by all the analyzed dermis cell types, including hematopoietic and non-hematopoietic. Unlike aqueous suspension, cream formulation also favored the maintenance of nanoparticles in the dermal architecture avoiding their dispersion and migration to draining lymph nodes via afferent lymphatics.

## Introduction

Nanomaterials, which size is included within 1–100 nm range, hold tremendous potential in biomedical applications thanks to the favorable combination of unique chemical and physical size-dependent properties [1,2]. In particular, nanoparticles can be designed in many different fashions and their clinical applications in drug delivery and diagnosis of human diseases are taken into consideration [3]. Among them, magnetite nanoparticles are ideal candidates as contrast agents for magnetic resonance imaging and magnetic force-assisted drug delivery systems [4]. It has been demonstrated several times that MNP are nontoxic at therapeutic dosages and the first MNP-based medical tools have already entered the marketplace [5] or are currently subjected to clinical trials [6].

Drug delivery nanosystems are meant to improve the biopharmaceutical properties of existing drugs that often exhibit a limited effectiveness in therapy. Such limitations include solid- and suspension-state instability, poor solubility and poor drug absorption that could lead to low bioavailability and insufficient targeting efficiency that could lead to unfavorable ratio between the amount of the administered drug and the concentration at the target tissue [7–9]. In order to optimize the loco-regional release of therapeutics, the topical route has been one of the most promising noninvasive delivery options, ameliorating patient compliance, improving the pharmacokinetics of degradable compounds and reducing frequently occurring side effects [10–12]. Nevertheless, drug topical administration remains a challenge in pharmaceutics because of the difficulties to adjust the skin penetration and to determine and reproduce the exact amount of drug reaching the skin layers at the desired depth [13,14]. In fact, the conventional transdermal drug delivery methods are limited by skin barrier properties according to the “brick and mortar model” (*stratum corneum*) that prevents excessive water loss and offers an efficient protective tissue against exogenous chemical, physical and mechanical stimuli and pathogens [15].

In this scenario, the preparation of high quality engineered colloidal nanoparticles loaded with drugs and modified with targeting molecules to improve the localized therapeutic effect represents a promising new strategy to develop a novel generation of therapeutic agents [16]. Despite at the moment only few studies have been reported on transdermal penetration of colloidal nanoparticles, cutaneous administration is expected to raise interest in the next future to achieve more efficient loco-regional delivery [17–19]. However, most attempts have mainly exploited invasive and expensive enhancement techniques or electrical methods to improve the penetration of nanoparticles in the healthy skin, with controversial conclusions [20]. So far, very few examples have been reported using human skin as a model for ultrastructural investigations [17].

To our knowledge, cream formulations have never been exploited to improve the absorption of nanoparticles. Water-in-oil (w/o) creams, which consist of small droplets of water dispersed in a continuous oily phase, can be used to improve the cutaneous absorption of pharmaceutical agents and to facilitate their penetration through the skin layers [21].

In the present work, we have synthesized MNP coated with a pro-functional amphiphilic polymer and used them as a colloidal aqueous suspension or formulated in a w/o cream. These nanoparticles, termed PMNP, were found to be colloidally stable both in aqueous fluids and in a w/o cream formulation and thus particularly appropriate to gain information on the destiny of nanoparticles. PMNP were used to study penetration in human skin samples by ultrastructural analysis and to assess nanoparticle distribution in immune cells of dermis in living mice. Despite mice skin is remarkably thinner than human, localized cells of innate immunity behave very similarly for this reason living mice represent a good model in order to investigate the interaction between colloidal nanoparticles and dermal cytotypes.

## Materials and Methods

### Ethics Statement

We declare that all *in vivo* experiments were performed using protocols approved by the University of Milano-Bicocca Animal Care and Use Committee. Protocols were approved by the Italian Ministry of Health under the protocol number 11–2012. Consensus of the Ethical Committee “Niguarda Cà Granda” Hospital, Milan was obtained for the use of fresh human skin samples.

### Mice and Reagents

All mice were bred on a C57BL/6 background and animals were housed under pathogen-free conditions. C57BL/6 mice were purchased from Charles River and were maintained in our animal facility at the University of Milano-Bicocca. Mice were housed in containment facilities of the animal facility and maintained on a regular 12:12 hour light:dark cycle with food and water ad libitum.

Fresh human skin samples were obtained by Bank Tissue after ethical committee consensus (Ethical Committee “Niguarda Cà Granda” Hospital, Milan). Cell culture medium and chemicals were purchased from EuroClone (Pero, Italy). All the antibodies (anti-CD11c, anti-CD11b, anti-CD19, anti-CD3, anti-CD45.2) used for fluorescence-activated cell sorting (FACS) analysis were purchased from BD Biosciences (San Diego, CA). For TEM images of the tissues, at the end of Franz Cell experiment, small portions of skin were fixed in 2.5% glutaraldehyde in 0.1 M phosphate buffer, pH 7.2 (Electron Microscopy Sciences, Hatfield, PA), for 2 h. After one rinse with phosphate buffer, specimens were post fixed in 1.5% osmium tetroxide (Electron Microscopy Sciences) for 2 h, dehydrated by 50, 70, 90, and 100% EtOH, and embedded in epoxy resin (PolyBed 812 Polysciences Inc., Warrington, PA).

#### Synthesis of PMNP and cream and suspension formulation

MNP were synthesized by solvothermal decomposition in octadecene from iron oleate precursor, as described previously [22]. MNP (10 mg) suspended in chloroform (5 mg mL^–1^) were transferred to water phase by mixing with a 0.5 M solution of an amphiphilic polymer (poly(isobutylene-alt-1-tetradecene-maleic anhydride)) (PMA, 136 μL) in 5 mL of sodium borate buffer (SBB, pH 12) [23]. The resulting PMA-coated nanoparticles (PMNP) were dispersible in aqueous media. For *in vivo* experiments green dye-labeled PMNP were synthesized using 5(6)-carboxyfluorescein diacetate N-succinimidyl ester (CFSE). After activation of the carboxylate groups of the PMA by 0.1 M EDC (6.5 μL), 0.05 M 2,2-(ethylenedioxy)-bis(ethylamine) (EDBE, 2.5 μL) was added and stirred 2 h. Next, nanoparticle dispersion was concentrated and washed twice with water. Subsequently, CFSE (1.8 mg dissolved in 180 μL of dimethyl sulfoxide) was added to 8 mg of nanoparticles, stirred 2 h and washed twice with water. The amount of CSFE onto PMNP was determined by measuring fluorescence emission at 518 nm (λ_ex_ 491 nm).

For PMNP suspension, PMNP as synthesized are concentrated in Amicon tubes (50 kDa filter cutoff) (Millipore Corporation, Billerica, MA) by centrifuging at 3000 rpm in order to obtain the final concentration of 8 mg mL^–1^ (5 × 10^14^ nanoparticles mL^–1^). The PMNP cream formulation was obtained by addition of 8 mg PMNP or PMNP-CFSE to 1 g of w/o cream (Essex cream, Shering-Plough) according to the method of progressive dilution.

#### Dynamic light scattering (DLS) and zeta-potential measurements

DLS measurements were performed at 90° with a 90Plus Particle Size Analyzer from Brookhaven Instruments Corporation (Holtsville, NY), working at 15 mW of a solid-state laser (λ 661 nm). Nanoparticles were dispersed in the solvent and sonicated in a S15H Elmasonic apparatus (Elma, Singen, Germany) before analysis. Final sample concentration used for measurements was 5 mg mL^–1^. Zeta-potential measurements were elaborated on the same instrument equipped with AQ-809 electrode and data were processed by ZetaPlus Software (Brookhaven Instruments Corporation, Holtsville, NY).

#### Transmission electron microscopy (TEM) of MNP and PMNP

For TEM analysis, MNP and PMNP were dispersed in hexane and water, respectively (50 μ g mL^–1^), and a drop of the resulting solution was placed on a Formvar/carbon-coated copper grid and air-dried. TEM images were obtained by a Zeiss EM-109 microscope (Oberkhochen, Germany) operating at 80 kV.

#### Skin preparation

A central punch biopsy of full skin thickness (epidermis, dermis and partially hypodermis) for each specimen was reduced for Franz Diffusion Cell (FDC) analysis. After the reduction the specimens (n = 2, for each 4 experiments) were placed in Petri dishes, epidermis side-up and dermis submerged in PBS (0.1 M, pH 7.4), then were thawed at room temperature (RT) till the experimental set up.

#### Histological analysis

A fragment of each skin biopsy was fixed in 4% buffered formaldehyde solution (0.1 M PBS, pH 7.4) for 5 h at RT, thoroughly washed with PBS, dehydrated using graded ethanol, and embedded in paraffin. Paraffin serial sections were prepared at a thickness of 5 μm, deparaffinized, and stained with haematoxylin and eosin. All sections were analyzed using a Leica microscope (DM2500) equipped with a digital camera (Leica, Wetzlar, Germany).

#### Skin penetration study with Franz Diffusion Cell

FDCs were maintained at 32°C with thermostated water in the jacket surrounding the cells. Receptor chambers were filled with 5 mL PBS and stirred continuously using a magnetic stir bar. Human skin (epidermis side-up, facing the donor chamber) was mounted into FDC interface and the PMNP formulations (saturated NP cream >10 mg cm^–2^ or solution >100 μ L cm^–2^, volume 1 mL) were applied to the donor chambers on the basis of an infinite dose test. All the experimental conditions were studied to simulate the physiological condition of temperature, pH and moisture. At specific time intervals (1-3-5-7-24 h), 500 μ L samples were withdrawn from the receptor chamber using a syringe and the same amount of fresh PBS was replaced in the same chamber at each time point. Statistical analysis was performed by means of two-tail t-student's test (Excel 2007, Microsoft, Redmond, WA), and the differences were considered statistically significant for p < 0.05.

#### Nanoparticles quantification by ICP analysis

For ICP-OES analysis, to 500 μ L samples, collected at specific times from the receptor chambers, were added 3 mL of aqua regia and, after 72 h, the samples were diluted with 7 mL of distilled water. All samples were measured in triplicate with Optima 7000 DV ICP-OES (Perkin Elmer, Waltham, MA).

#### Skin fixation protocol for SEM and TEM analysis

After the fixation, longitudinal skin sections of 70–80 nm thicknesses were obtained by perpendicular cutting to avoid the tissue disruption and were examined by means of a transmission electron microscope (Zeiss EM109) (Zeiss, Oberkochen, Germany) operating at 80 kV. For SEM images of the tissues, at the end of Franz Cell experiment, small portions of skin were fixed in 2.5% glutaraldehyde in 0.1 M phosphate buffer pH 7.2 (Electron Microscopy Sciences, Hatfield, PA), for 24 h. After one rinse with phosphate buffer, specimens were dehydrated by 10, 25, 50, 75, and 100% EtOH, and dried with hexamethyldisilazane. The samples are carefully mounted on a stub and coated with a very thin film of Agar Auto Sputter Coater (Assing, Monterotondo, Italy) before SEM examination with Leica S420 Microscope operating at 15 Kv.

#### Isolation of skin cells

Cells were isolated as previously described [24]. Briefly, skin was isolated and digested for 45 min in a cocktail containing collagenase XI, hyaluronidase and DNase. 10% fetal bovine serum (FBS) was added to stop the reaction and cells were then stained to assess the percentage of different cell populations.

#### Flow Cytometry

Single cell suspensions were washed with ice-cold PBS and stained with the appropriate antibody for 30 min on ice, followed by ice-cold PBS washing. Staining was assessed with a FACS Calibur (Becton Dickinson).

## Results and Discussion

### Colloidal nanoparticles synthesis and characterization of and formulation in a w/o cream

**Fig 1:**
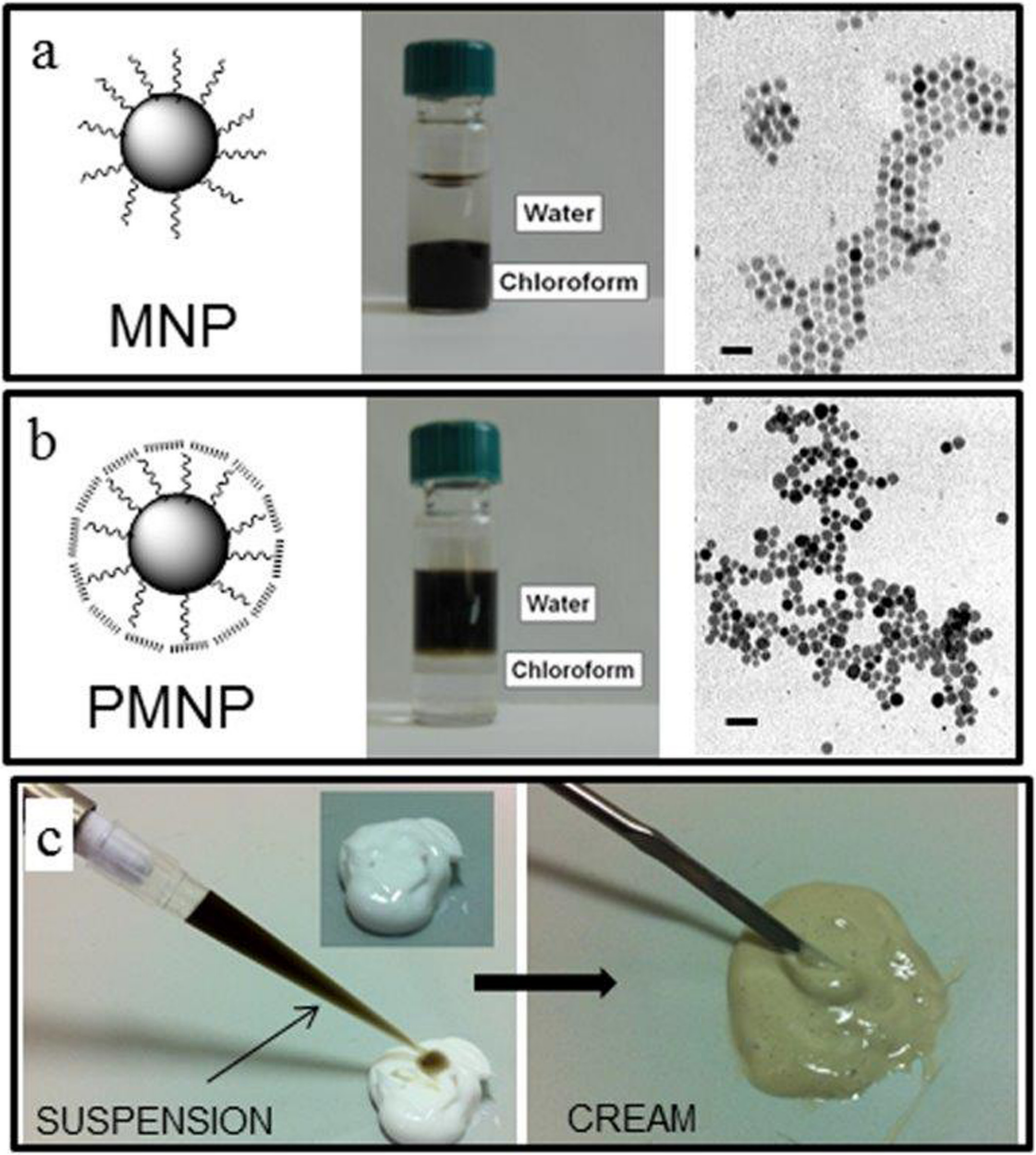
Fe_3_O_4_ nanoparticles (MNP, a) synthesized in organic solvent and transferred to a water solution using PMA amphiphilic polymer (PMNP, b). MNP and PMNP were highly monodisperse in size as it is shown by TEM images (scale bars = 40 nm,). Part of the highly concentrated PMNP suspension (8 mg mL^–1^) was incorporated in a w/o cream (0.8 wt % concentration) (c).

Hydrophobic magnetite nanoparticles (MNP) were synthesized from organometallic precursors by solvothermal decomposition in octadecene [22]. The high temperature and the presence of stabilizing surfactants provided an optimal crystal nucleation and growth resulting in highly uniform and monodisperse nanoparticles with 12.0 ± 1.2 nm average diameter, as measured by TEM, that were suspended in chloroform by virtue of the oleate surfactant coating. A TEM image of MNPs is shown in Fig 1A. This feature was further confirmed by dynamic light scattering (DLS) analysis, which resulted in a hydrodynamic diameter of 19.0 ± 1.3 nm in hexane. To produce a stable suspension in a physiological environment, a water phase transfer of nanoparticles was needed. To this aim, MNP were coated with an amphiphilic polymer (PMA), obtained by reacting poly(isobutylene-*alt*-1-tetradecene-maleic anhydride) with an amount of dodecylamine sufficient to react with 75% of anhydride groups (Fig 1B) [24]. In MNP coating process, the hydrophobic alkyl chains intercalated between those of oleic acid, which acted as a surfactant (PMNP, 12.2 ± 1.8 nm, TEM measured diameter). The resulting nanoparticle suspension exhibited important advantages including: 1) a stable colloidal dispersion both in aqueous solution and physiological buffer; 2) the polymer coating can be easily functionalized with drugs or targeting molecules for future therapeutic applications; 3) the amphiphilic coating was expected to improve the nanoparticle penetration through both hydrophobic and hydrophilic skin layers; 4) the amphipathic character of the composite nanomaterial was suitable to obtain a homogenous cream nanoformulation. In this way, we developed a nanoparticle scaffold that could be formulated both in suspension (even at a PMNP concentration of 8 mg mL^–1^) and in w/o cream, which might be useful for topical application. As expected, PMNP nanoparticles coated with PMA appeared a bit larger than MNP by themselves at DLS analysis (23.2 ± 2.0 nm) but were still stable and monodisperse in size. Beyond, the ζ-potential analysis showed a strongly negative surface charge in water (ζ = –55.98 ± 3.18 mV). To evaluate the potential of PMNP for future application as magnetic resonance imaging contrast enhancers, the value of decreasing concentrations of PMNP in water was 186 mM^–1^s^–1^ at 0.47 T. This value is higher than the negative contrast power of agents currently available in approved clinical diagnostic practice (*r*_2_ of Ferumxytol is 83 mM^–1^s^–1^ at 0.47 T). For *in vivo* studies, CFSE was conjugated to PMNP using the bifunctional diamino-linker 2,2-(ethylenedioxy)-bis(ethylamine) (EDBE) previously activated by *N*-(3-dimethylaminopropyl)-*N*′-ethylcarbodiimide (EDC). Both labeled and unlabeled PMNP were formulated in a cream, using the method of progressive dilution, in order to obtain a homogeneous preparation (Fig 1C). The maximum amount of PMNP included in 1 g of cream for biological experiments was 8 mg, used to test *in vitro* diffusion and *in vivo* absorption. Physical stability of the cream containing PMNP was also evaluated in order to test the stability of the final product. The organoleptic properties did not show any changes over two years examination.

### Investigation of nanoparticle penetration through the human skin.

**Fig 2:**
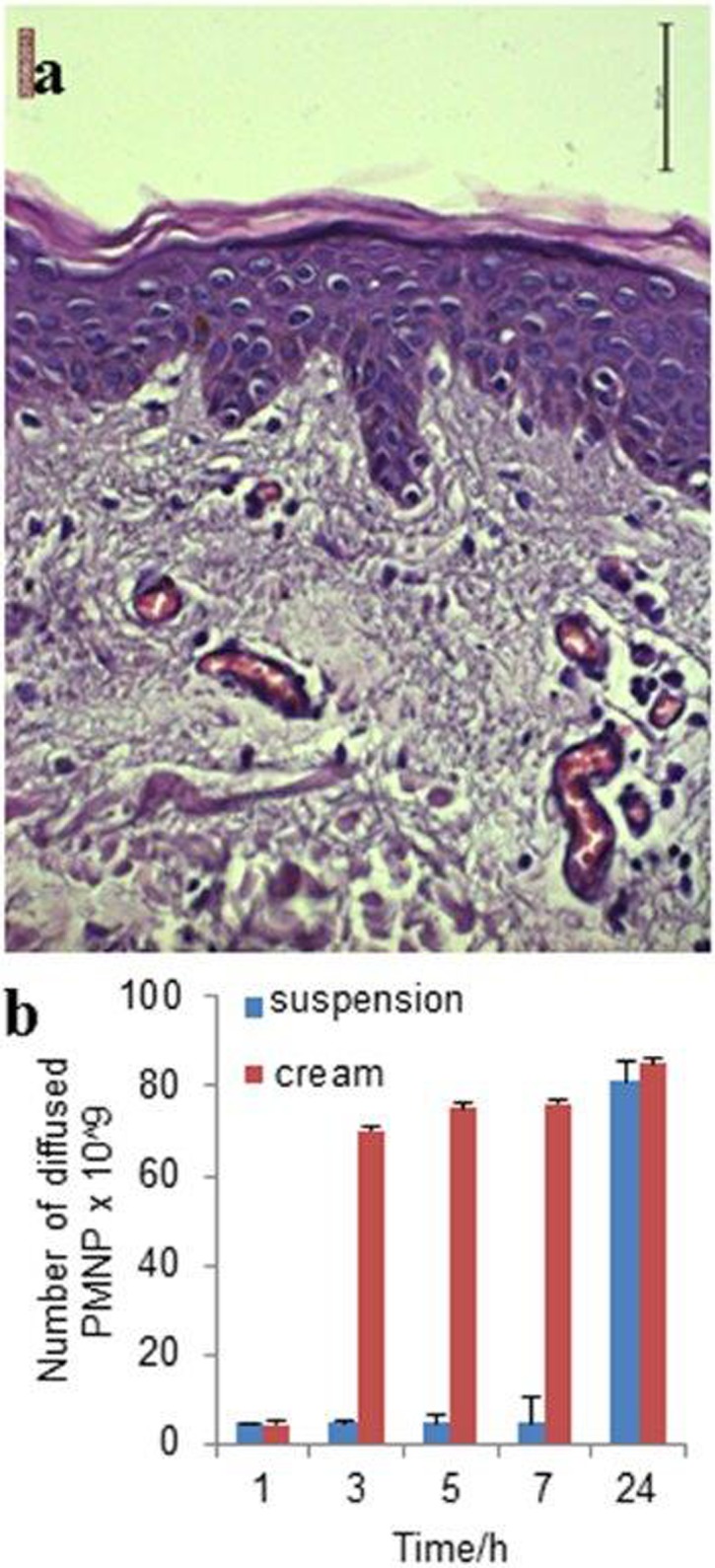
Histological microphotograph of normal human skin section. Haematoxylin and eosin staining (original magnification 40×) (a). *In vitro* diffusion studies of PMNP colloidal suspension or cream in human skin were carried out using Franz diffusion cells and diffused PMNP were quantified by ICP-OES analysis (b).

In order to investigate nanoparticles penetration applied with both types of formulations, through all skin layers, *in vitro* skin permeation studies were carried out using normal human skin in FDCs. Before the penetration study, a histological analysis was performed in parallel on human skin biopsies to verify the morphological integrity of all skin layers. Histological examination of haematoxylin and eosin-stained skin sections revealed a regular histological appearance of epidermis and dermis (Fig 2A). A deep monolayer of well-preserved cubical and cylindrical cells (basal layer), an intermediate spinous layer, an upper granular layer, and outermost stratum corneum were always observed. Compact papillary and reticular dermis filled with blood vessels were also maintained.

After histological validation, nanoparticle permeation in different skin layers was analyzed by TEM and SEM. Receptor chambers were filled with PBS (5 mL) and PMNP were applied to the donor chambers. Samples of receptor fluid were taken at various time intervals (1-3-5-7-24 h) and the relevant PMNP concentrations in the receptor fluid were assessed measuring ICP-OES values normalized with values obtained by control samples. Next, nanoparticle permeation in different skin layers was analyzed by transmission and scanning electron microscopies. Fig 2B, at the end of skin exposure time (24 h), shows no significant difference between cream and suspension administration was observed in the amount of diffused nanoparticles through the human intact skin (p>0.05); on the other hand, at previous sampling times (3, 5 and 7 h), the differences were significant (p = 3.6*10^–9^ at 3 h, p = 4.0*10^–8^ at 5 h, p = 1,8*10^–6^ at 7 h oppure p = 3.6*10^–9^, p = 4.0*10^–8^ and p = 1,8*10^–6^ respectively). This result can be explained by the physico-chemical properties of PMNP nanoparticles, which allowed the penetration from side to side of the skin barrier. In fact, the superficial amphiphile-like behavior of the polymer might favor the optimal permeation of both the hydrophobic and hydrophilic layers. The application of PMNP w/o cream formulation gave an accelerated and homogeneous nanoparticle permeation resulting in a reduction of the penetration and diffusion time, probably allowed by skin hydration and achieved by cream application. Moreover, the occlusive effect of the cream prevented the water evaporation and the loss of moisture promoting the nanoparticles penetration. Indeed, PMNP suspension required 24 h to equilibrate the penetration kinetics through the dermis.

### Electron microscopy analysis of skin penetration

**Fig 3:**
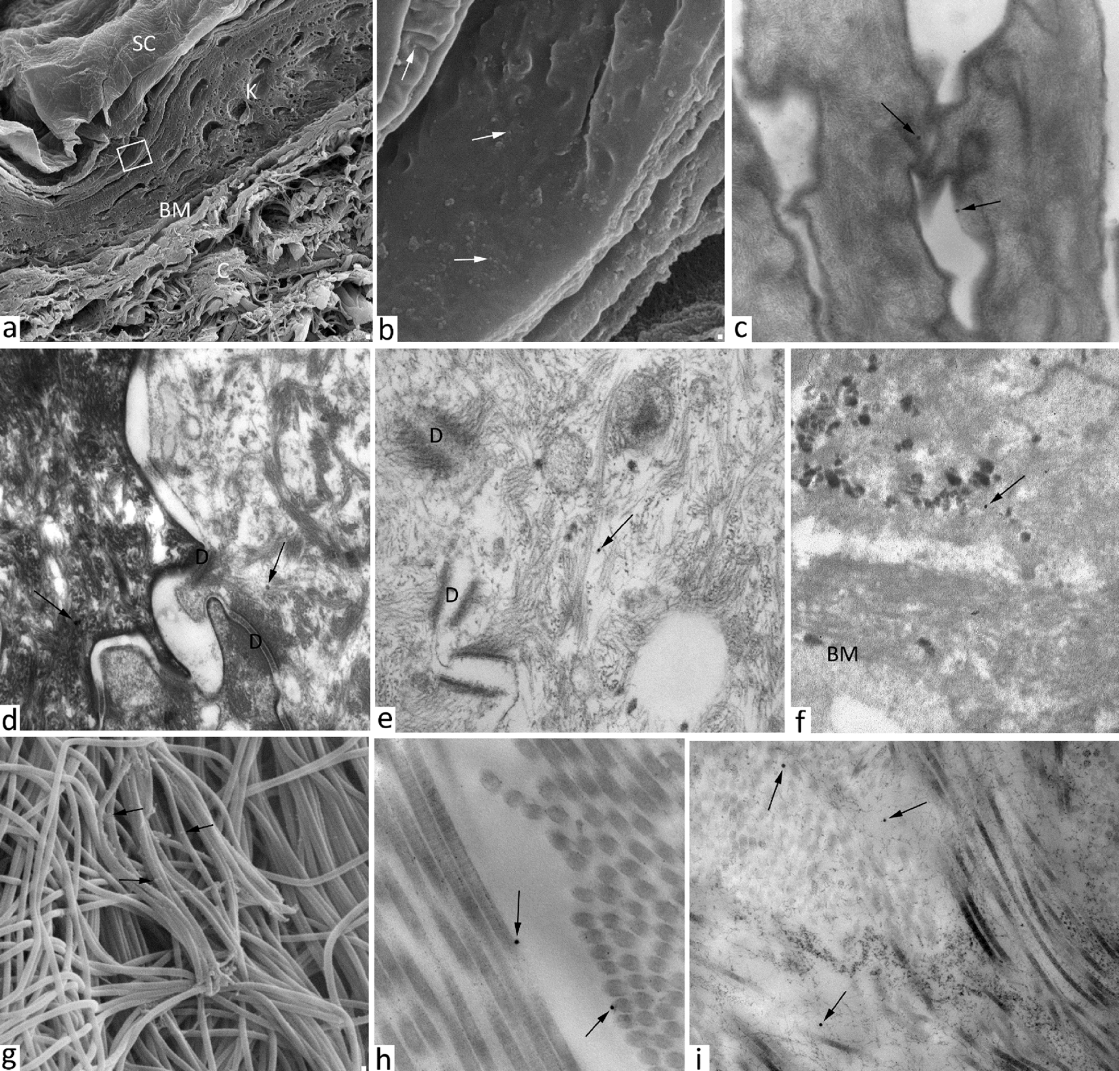
Images of skin samples treated with PMNPcream or suspension obtained with transmission and scanning electron microscopy. Selected micrographs show (a) SEM image of transversal skin section treated with PMNP solution for 24 h, SC = *stratum corneum*, K = keratinocytes, BM = basal membrane, C = collagen, (b) high magnification of Fig “a”, PMNP are detected on the surface of the keratinocytes (white arrows). TEM images of PMNP nanoparticles (black arrows) inside the corneocytes of the skin treated with cream (c, d) or suspension (e, f) PMNP nanoparticles in proximity of the basal membrane, D = desmosomes. g), h) and i) photomicrographs of PMNP nanoparticles (black arrows) intercalated between the collagen fibers of the dermis, g) SEM image h) and i) TEM images.

There is evidence that the major constrain for skin penetration is due to the *stratum corneum* barrier [12]. Three possible permeation routes have been identified including intracellular, intercellular, and follicular pathways. In principle, the latter should be considered the easiest way to occur for large molecules, due to the obvious preferential pathway along the follicular channels. However, in human skin, hair distribution represents only a small skin area fraction [14]. On the other hand, it is more difficult to discriminate between intracellular and intercellular routes. Fig 3 shows PMNP intradermal delivery across the intact skin layers observed by electron microscopy. Nanoparticles can be clearly distinguished in all skin layers, from the surface to deeper levels, both in cream and suspension formulations. No significant differences were detected between the two formulations in terms of permeation mechanism and amount of penetrated nanoparticles after 24 h. PMNP amphiphilic polymer coating confers nanoparticles both a hydrophobic character, useful for the penetration of waterproof corneocyte-based *stratum corneum*, and a hydrophilic propensity required for deeper viable epidermis permeation. TEM images suggest that nanoparticles penetrated mainly through intercellular pathway, as numerous nanoparticles can be distinguished across epidermis cells and through the whole skin thickness (Fig 3A–3C). However, the presence of PMNP among corneocytes provided direct evidence that the intracellular penetration pathway also occurred both in cream and in suspension (TEM images in Fig 3D and 3E). PMNP were also observed close to the basal membrane (Fig 3F). After epidermis penetration, nanoparticles reached the dermis layer, where they were detected in proximity to the collagen fibers (Fig 3G–3I). A large number of nanoparticles could be recovered in the dermis in higher concentration than expected from previous reports, probably due to the contribution of the amphiphilic polymer coating [20]. Another important observation was that, analyzing several dermis sections by TEM/SEM, nanoparticles maintained their individuality and did not aggregate. This result was confirmed by DLS analysis of the solution collected in the receptor chamber of the FDC, in which nanoparticles in the 35–65 nm size range were recovered. This is consistent with the assumption that after passing through different skin layers, the biological identity of nanoparticles, including the formation of a thick protein corona, was expected to change by adsorbing various biological materials on their surface [7,24]. As control, we found the similar values measuring PMNP size after incubating them in fetal bovine serum media.

### Interaction with skin-localized cells of innate immunity in living mice

**Fig 4:**
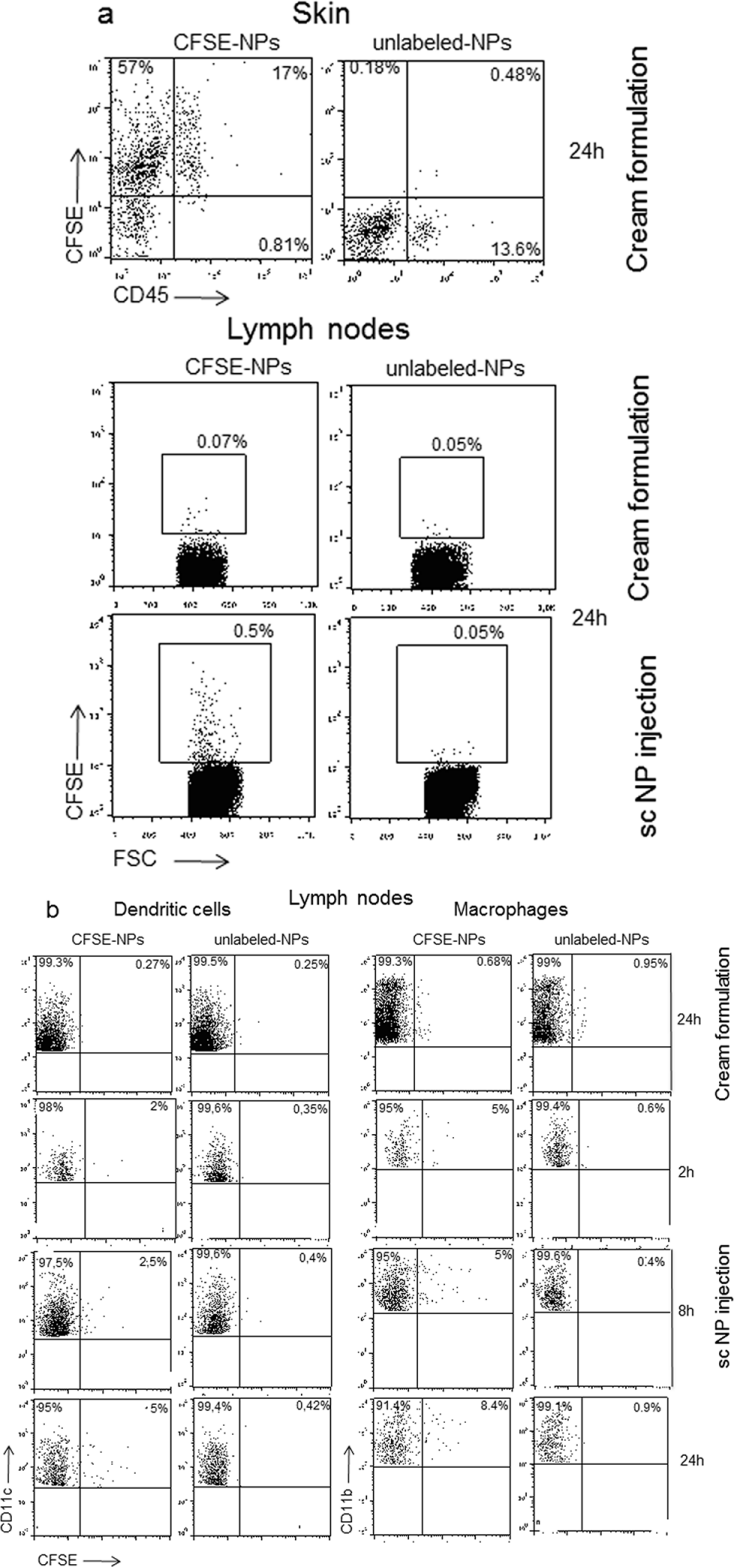
Cytofluorimetric analysis showing PMNP nanoparticles uptake by mouse skin and lymph node cells. PMNP suspension (a, upper panels). Skin CD45-positive and negative cells showing CFSE incorporation. Note that most of the skin cells uptake PMNP nanoparticles administered with the cream formulation. (a, lower panels) CFSE-positive cells in the lymph nodes of mice that received PMNP nanoparticles via cream formulation or via sc administration. Note that only with sc PMNP administration, nanoparticle-positive cells can be detected in the draining lymph nodes. (b) Lymph node macrophages and dendritic cells, identified as CD11b- and CD11c-positive cells respectively, showing CFSE incorporation. Note that only when PMNP are administered sc, CFSE positive macrophages and dendritic cells can be detected in the lymph nodes.

**Fig 5:**
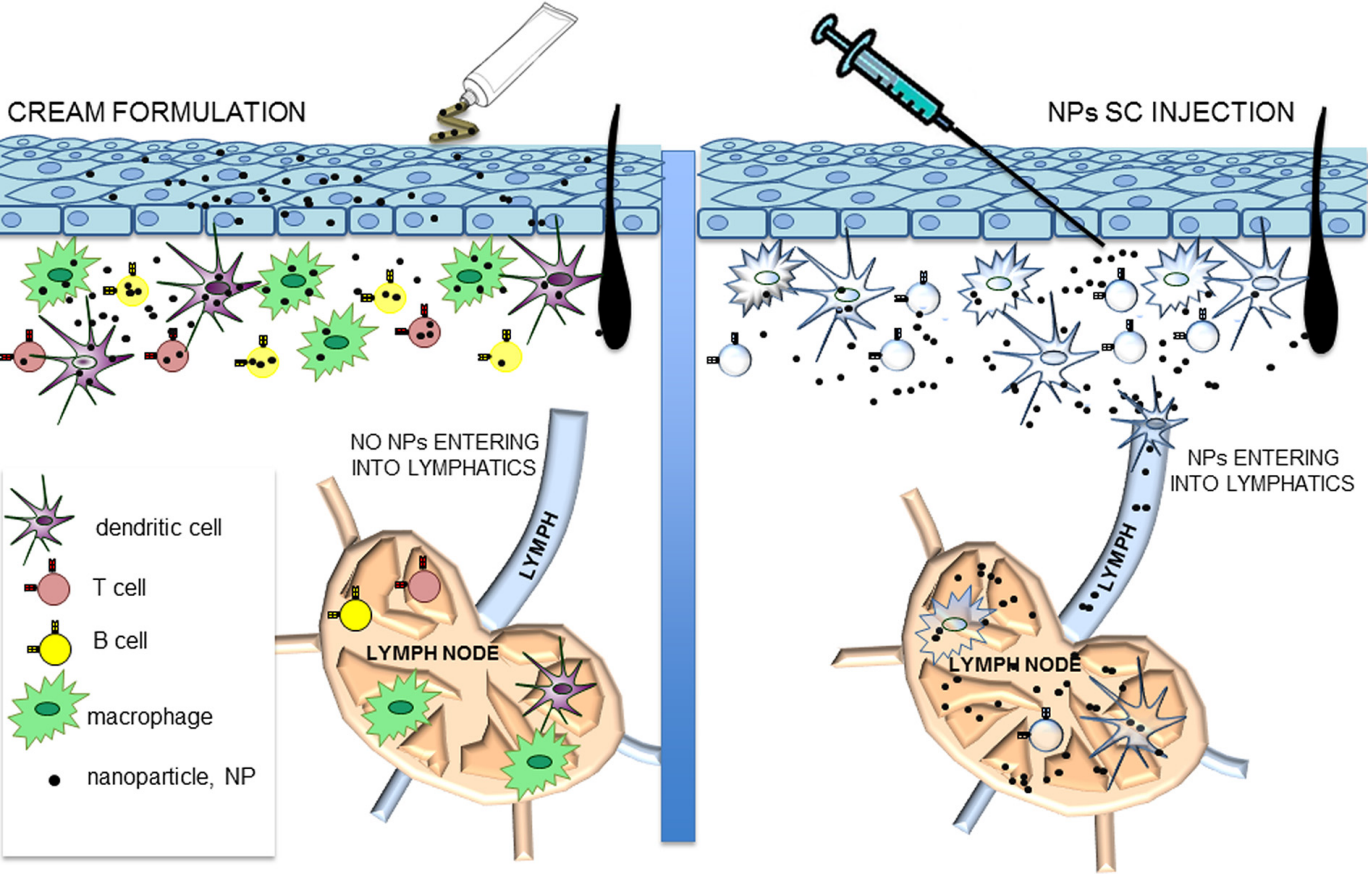
Fates of nanoparticles depending on the route of skin administration. Nanoparticle administered in a cream formulation are taken up by all the skin cell types and do no reach the draining lymph nodes. Nanoparticle administered with a sc injection in aqueous suspension are efficiently transported to the draining lymph nodes.

Nanoparticles incorporation by cutaneous cells was evaluated in mouse models. Cream formulations of CFSE-conjugated PMNP were applied to the skin of previously shaved C57BL/6 mice (1 cm^2^). Mice were sacrificed 24 h later and treated skin was recovered and analyzed by flow cytometry after having obtained single cell suspensions. Draining lymph nodes were also analyzed to evaluate the possible transport of nanoparticle via the lymph. Skin cells of hematopoietic and non-hematopoietic origin were identified as CD45-positive and negative, respectively. As shown in Fig 4, both populations contained fluorescent cells, indicating that both groups of cells could incorporate PMNP nanoparticles. Since no positive staining could be observed in the draining lymph nodes (Fig 4A) interestingly, nanoparticles administered with cream formulations remained confined to the skin. It is known that sub-cutaneously (sc) inoculated substances reach the draining lymph nodes by entering the afferent lymphatics free or associated with dendritic cells [25]. In order to double check the detection of nanoparticle-positive cells in the lymph nodes, if present, we injected CFSE-conjugated PMNP sc in an aqueous suspension. As Fig 4A clearly shows, after sc administration, CFSE-positive cells could be clearly detected in the draining lymph nodes. Since macrophages and dendritic cells are the cells that preferentially uptake particulate antigens inside the lymph nodes, we also analyzed these two cell types in mice that treated with PMNP cream or PMNP in aqueous solution. In the draining lymph nodes of mice that received PMNP nanoparticle via sc administration CFSE-positive macrophages and dendritic cells could be detected starting from 2 h after treatment. On the other hand no positive cells could be detected in the lymph nodes of mice treated with PMNP cream (Fig 4B). This data suggests that cream formulations, thanks to the high lipidic concentration, permeabilize different cell types, including T and B-cells, macrophages, dendritic cells and mast cells, and non-hematopoietic cells (CD45-negative cells), and entrap the nanoparticles avoiding their dispersions. Otherwise, not being entrapped in a lipidic film, nanoparticles freely administered in aqueous suspensions have easy open access to the afferent lymphatics and to draining lymph nodes (Fig 5). These results indicate that cream formulations represent the ideal solution when a topical localized treatment is needed and different cell types have to be targeted.

## Conclusions

In summary, in the present work we developed colloidal nanoparticles composed of an iron oxide nanocrystal core coated by a tightly bound amphiphilic polymer, formulated in a w/o cream able to permeate and penetrate the human skin. The same nanoparticles were also used for biological experiments in colloidal suspension as control. *In vitro* permeation analysis, carried out through intact human skin using Franz Diffusion Cell exhibited an increased, faster and more regular penetration of nanoparticles when formulated in cream in comparison to nanoparticle deposition as aqueous suspension. This makes nano-cream very promising for application in local therapies. However, probably due to the amphiphilic character of the polymer coating, nanoparticles formulated in suspension exhibited an intrinsic enhanced propensity to penetrate through the different human skin layers as well as results reported in previous literature [26]. *In vivo* treatment of living mice with these nanoparticles showed their uptake by the dermal resident immune cells. In particular, when administered in the form of nano-cream suspension, nanoparticles remained confined within the thin skin layers of mice, as no migration to the draining lymph nodes could be observed. The results obtained in this study offer promise in the development of nano-cream for the rapid targeting of bioactive agents and drugs in the skin barriers. Such molecules could be encapsulated in the hydrophobic shell or covalently linked to the nanoparticle surface, supporting the topical route as a convenient alternative to the parenteral administration of anti-inflammatory and cytotoxic nanoconjugate drugs reducing systemic effect of toxic agents.
